# The Human Brain; Its Structure, Physiology, and Diseases, &c.

**Published:** 1848

**Authors:** 


### ARTICLE II.

*The Human Brain: its Structure, Physiology, and Diseases; with a Description of the Typical Forms of Brain in the Animal Kingdom.* By Samuel Solly, F.R.S., Senior Assistant Surgeon to St Thomas Hospital, and Lecturer on Clinical Surgery, &c., &c. From the second London Edition: with one hundred and eighteen wood engravings. pp. 496, 8vo. Philadelphia: Lea & Blanchard. 1848. (From the publishers—for sale by Joseph Keene, Jr., Chicago.)

This is a re-print of an English work without American notes, comments, or additions; in other words, it is without an editor. It is a thing well enough to try a book on its own merits occasionally, in which case, if it is popular, there will be less difficulty in awarding the meed of praise for its success. It is often extremely difficult to say whether the work itself and the reputation of the author bears up the name of the editor, or whether the editor enhances the value and reputation of the work.

In reference to the character of the work before us, we can only say, that from reading part of it, and glances at the arrangement of the subject in the remaining portion, we believe it to be excellent.

The comparative anatomy and physiology, the minute examination of the structures, and the close enquiry into the functions of the different parts of the brain and nerves, in the first part of the work, are highly interesting; and from the amount of observation and research here manifested, we feel justified in expecting to find laid down in the subsequent more practical part of the work, in which the diseases of the brain and their treatment are considered, a clear pathology and correct practice. [E.

